# On Insufficient Vaccination

**Published:** 1872-03

**Authors:** Wm. Henry Cumming

**Affiliations:** Atlanta, Georgia


ATLANTA

### MEDICAL AND SURGICAL JOURNAL.

NEW SERIES.

VOL. IX.—MARCH, 1872.—NO. 12.

### ORIGINAL COMMUNICATIONS.

### ON INSUFFICIENT VACCINATION.

BY WM. HENRY CUMMING, M.D., ATLANTA, GEORGIA.

"In order that persons should have from vaccination the fullest protection against small-pox which it is capable of imparting, it is necessary that the vaccination should not only be perfect in character, but that it should be sufficient in amount—that the system should not only be infected, but that it should be well-infected. By observations of unsurpassable interest and value, made for upwards of thirty years at the Small-Pox Hospital, it has been demonstrated that the extent to which small-pox, if it should be contracted at all after vaccination, is modified by vaccination, is determined by the character and number of the cicatrices; that it is in the exact ratio of the excellence and completeness of the vaccination, as determined by these tests. Persons whose vaccination has resulted in their having one genuine vesicle, and one only, are, as a class, much less protected than those who have had two; those who have had two than those who have had three, etc.; and the protection against fatal small-pox which is afforded by four or more genuine vesicles is almost absolute." (Vide Seaton's Hand-Book of Vaccination, p. 242.)

There is a prevalent belief among our people, (and, I may safely add, among the members of the medical profession), that, with reference to their liability to contract small-pox, the entire population may be divided into two great classes—the vaccinated and the unvaccinated. The result of this belief is, that any one who has been vaccinated is deemed to be protected, so far as vaccination can protect. My object in the present article is to show that this belief is erroneous, and that many (may I not say most?) vaccinated persons are far less protected than they suppose—much less secure than they might have been.

And now, in the very first place, lest it should be for a moment supposed that my purpose is to discredit vaccination, let me here express my conviction, (which is that of the highest authorities), that *vaccination, performed in the best manner*, is almost, if not quite, as protective as small-pox itself. The following extract from the article on "Vaccination," in Reynold's System of Medicine, affords most valuable information on this point:

"The records of the Royal Military Asylum at Chelsea show, that of 5,774 boys admitted into that institution in the course of the forty-eight years, ending December, 1851—of whom 1,950 had, on admission, marks of small-pox, and 3,824 either had marks of vaccination or were, on admission, vaccinated—6.15 per thousand of the former and 7.06 per thousand of the latter contracted small-pox subsequently during their residence in the Asylum."

The protective power of small-pox here seems to be to that of vaccination inversely as 6.15 to 7.06 or directly as 7.06 to 6.15, which is equivalent to a superiority of small-pox to vaccination of only 0.91 per thousand, or 0.091 per hundred—not quite one-tenth of one per cent. It will be noticed, that of these 5,774 boys, one-third have marks of small-pox; in the year 1866, an inspection of the school-children in London showed only one in forty to be thus marked. So great has been the influence of the extension of vaccination during the years intervening between 1800 and 1860.

The army returns for the years 1859–1862 also clearly show the protective power of vaccination:

| YEAR. | No. Troops in United Kingdom. | Cases of Small-Pox. | Deaths. | Per 10,000. | |
|---|---|---|---|---|---|
| | | | | Cases. | Deaths. |
| 1859 | 71,715 | 175 | 7 | 24.3 | 0.97 |
| 1860 | 85,443 | 140 | 9 | 16.8 | 1.05 |
| 1861 | 88,955 | 51 | 4 | 5.9 | 0.45 |
| 1862 | 78,173 | 64 | 4 | 8.1 | 0.51 |

The two classes of facts just cited (in a military asylum and in army garrisons) show the protective power of vaccination combined with careful isolation of the sick, so as to give time for the protection of the others. But in civil practice, in families, and especially among the poor, the protective power of vaccination is perhaps still more fully shown. Here, where the children occupy the same house—often the same room, sometimes the same bed—the admirable results of vaccination are most clearly seen. In one town, among three hundred persons thus exposed, two hundred and fifteen were unprotected, and ninety-one had been vaccinated. Of the two hundred and fifteen, only fifteen escaped the disease; two hundred contracted it, of whom forty-six died, or almost one-fourth. Of the ninety-one vaccinated, only two had the disease, in its modified form, and both recovered.

Among unvaccinated persons affected with small-pox, the death-rate is from twenty-five to thirty-three per cent.—one-third or one-fourth of the cases usually proving fatal; among the vaccinated, the rate is much lower, being, on the whole, about six or seven per cent., or one death in fifteen cases.

The observations made by Marson, at the Small-Pox Hospital in London, on 15,000 cases treated there during thirty years, show conclusively that the degree of modifying power is in the exact ratio of the excellence and completeness of the vaccination, as shown by the cicatrices; in other words, that it is directly as the amount of vaccine-marking and as the character of the marks. This may be readily seen from the following table:

| CLASSIFICATION OF PATIENTS AFFECTED WITH SMALL-POX. | No. deaths per ct. in each Class | Safety from Death, if affected with Small-Pox. | | | | |
|---|---|---|---|---|---|---|
| A.—Unvaccinated........ | 37 | 1 | | | | |
| B. Stated to have been vaccinated, but bearing no mark......... | 23.57 | 1.57 | 1 | | | |
| C.—Having one bad scar. | 11.91 | 3.10 | 1.97 | 1 | | |
| D.—Having two bad scars | 8.34 | 4.43 | 2.82 | 1.43 | | |
| E.—Average vaccination. | 6.50 | 5.69 | 3.62 | 1.83 | 1 | |
| F.—Having one good scar | 3.83 | 9.66 | 6.15 | 3.11 | 1.70 | 1 |
| G.—Having two......... | 2.32 | 15.94 | 10.15 | 5.13 | 2.80 | 1.65 |
| H.—Having three........ | 1.95 | 18.97 | 12.08 | 6.11 | 3.33 | 1.96 |
| I.—Having four or more. | 0.55 | 67.27 | 42.84 | 21.65 | 11.82 | 6.97 |

From this table, we see that those most fully protected (Class I) have so little prospect of dying from small-pox, that if two hundred were to take the disease, only one would die; and when we combine with this the other fact, that less than four per cent. of this class are likely to contract the disease, we can readily compute the liability of this class to die from this cause, and find it to be only one in 5,000.

The mortality among those forming the second class (B) is only two-thirds of that among the altogether unvaccinated. The operation in these cases, although so ill-performed as to leave no visible trace, has had an appreciable result: it has saved one life out of three. Of those having two bad scars (Class D), more than three-fourths (twenty-eight out of thirty-seven) have been saved.

We see, too, how low is the average vaccination in England, or rather how low it has been during a great part of this century. It ranges in efficiency between that of two bad scars and one good scar; and yet it has saved nearly five-sixths (thirty out of thirty-seven) of those attacked. But it is on examining the last four classes (F, G, H, I,) that we see the importance of *full* vaccination. Is it possible that so many are in the Class F, when three or four additional punctures (requiring not one minute of time) would place them in a position of *seven-fold* security? These truths (if truths they be) are worthy of the most serious attention on the part of parents as well as of physicians. No physician should fail to give his patients the utmost attainable protection from this loathsome and fatal disease; no parent should fail to secure it for his child.

Let us look at two hundred persons affected with small-pox. If entirely unprotected, one hundred and twenty-six will recover, and seventy-four will die; if most imperfectly vaccinated (so as to leave no mark that can be recognized), one hundred and fifty-three will recover, and forty-seven will die; If the vaccination be complete, one hundred and ninety-nine will recover, and only one will die; with the ordinary average vaccination of England, one hundred and eighty-seven will recover, and thirteen will die.

In order to set forth these facts with the utmost simplicity and clearness, the subjoined table is introduced:

| | In One Thousand Cases of Small-Pox. | | |
|---|---|---|---|
| | Recoveries. | Deaths. Total. | Deaths. Preventible. |
| A.—Unvaccinated | 630 | 370 | 365 |
| B.—Vaccinated (no mark) | 765 | 235 | 230 |
| C.—Having one bad scar | 881 | 119 | 114 |
| D.—Having two bad scars | 917 | 83 | 78 |
| E.—Average vaccination | 935 | 65 | 60 |
| F.—Having one good scar | 962 | 38 | 33 |
| G.—Having two good scars | 977 | 23 | 18 |
| H.—Having three | 981 | 19 | 14 |
| I.—Having four or more | 995 | 5 | 0 |

We have thus far considered the protection afforded by the various kinds and degrees of vaccination in those cases where the infection of small-pox has occurred. But does not vaccination preserve from the infection itself? and if so, to what extent? and are there degrees of protection against the infection as there are against death in cases of the disease incurred?

An answer to these questions may be found in the results of certain inspections of children in schools, work-houses, etc., in England. Of one thousand unvaccinated children, three hundred and sixty bore marks of small-pox—more than one-third. But these three hundred and sixty living children probably represent a much larger number. As one-third of the cases of this disease in the unvaccinated usually prove fatal, we are justified in supposing that these three hundred and sixty are the survivors of five hundred and forty cases: that is, that of eleven hundred and eighty children, six hundred and forty had escaped the disease, three hundred and forty had recovered, and 186 had died; and of those who had recovered, many were so marked (says the Inspector) as to be really hideous to look at, and in several, the disease had left permanent blindness or deafness.

In marked contrast to this gloomy picture is that of the vaccinated, who furnished, in a thousand, only two cases bearing any traces of small-pox. Instead, therefore, of three hundred and sixty cases, they show two, or only one case to one hundred and eighty of the unvaccinated; or, more exactly, if we add to the number of the unvaccinated those who have died, we have the contrast of two (per thousand) to four hundred and fifty-seven, or of one to two hundred and twenty-eight.

| Classification of Children Examined. | Proportion marked with Small-Pox per 1,000 Children in each Class respectively. |
|---|---|
| 1. Having no vaccine marks | 360 |
| 2. Having one vaccine mark | 6.8 |
| 3. Having two vaccine marks | 2.49 |
| 4. Having three vaccine marks | 1.42 |
| 5. Having four or more | 0.67 |

Safety from death in the class having one good scar (vide F, in our second table) is, to the security enjoyed by those having four or more, as 0.55 to 3.83, or as one to seven. Safety from an attack of small-pox severe enough to leave traces is seen, from this last table, to be in the same classes (2 and 5) as 0.67 to 6.8, or as 1 to 10.2. If this last relation truly represents the probabilities of having small-pox in any form, we may proceed one step farther. If, with only one scar, the probability of having the disease is ten-fold greater than with four or more scars, and at the same time the probability of death (if the disease is taken) is seven-fold greater, then the necessary inference is that the former class is *seventy* times more in danger of dying from small-pox than the latter; and if this be true, the more complete and *sufficient* vaccination might well receive seventy times the care, and would be well worth seventy times the pecuniary reward.

Well may we conclude this article in the words of the author whom we have cited in our opening paragraph: "These observations form the basis on which vaccination should always be conducted. No practitioner, it may be safely said, will have done his duty in any case in which he is called upon to vaccinate, unless, besides all requisite precautions with regard to the genuineness of the lymph employed, and the means of insuring success, he has also taken care to vaccinate *sufficiently*—*i. e.*, to produce, so far as in him lies, four or five genuine, good-sized vesicles, such as result from separate punctures, or, if vaccinating otherwise than by separate punctures, to produce equivalent local results."

Having thus shown that there are various degrees of efficiency in vaccination, we hope to indicate, in a future article, the conditions of the highest success.

